# Gastrointestinal symptoms - an illness burden that affects daily work in patients with IBS

**DOI:** 10.1186/s12955-019-1174-1

**Published:** 2019-07-01

**Authors:** Åshild Faresjö, Susanna Walter, Anna-Karin Norlin, Tomas Faresjö, Michael P. Jones

**Affiliations:** 1 Department of Medicine and Health, Community Medicine, Faculty of Medicine and Health Sciences, Linköping University, SE-581 83 Linköping, Sweden; 2 Department of Clinical and Experimental Medicine, Division of Gastroenterology, Faculty of Medicine and Health Sciences, Linköping University, Linköping, Sweden; 3 Psychology Department, Macquarie University, Sydney, NSW Australia

**Keywords:** IBS, Disease burden, Sense of coherence, Confidence in public health

## Abstract

**Background:**

Irritable Bowel Syndrome (IBS) is a chronic gastrointestinal disorder characterised by recurrent abdominal pain and disturbed bowel habits and unclear aetiology. IBS is also associated with psychosocial factors, impaired quality of life and lost work productivity. This study sought to determine whether the association between IBS and lost work productivity might be accounted for by poor coping strategies and loss of confidence in the healthcare system.

**Methods:**

Case–control design was employed sampling IBS and non-gastrointestinal (non-GI) primary healthcare seekers in a defined region in Sweden. Non-GI patients were of similar age and sex distribution to the IBS patients. Questionnaires applied in this study included instruments designed to measure confidence in the social security system and in the community, as well as questions about whether gastrointestinal problems might affect working life and Sense of coherence (SOC) questionnaire. The study’s primary hypothesis was evaluated via an a priori path model.

**Results:**

Statistically significant differences were found between IBS cases (*n* = 305) and controls (*n* = 369) concerning abdominal pain or discomfort affecting everyday performance at work (*p* <  0.0001). IBS cases also showed significantly lower (*p* = 0.001) confidence in public healthcare. The study’s hypothesis was supported with the finding of a statistically significant indirect association via poor coping strategies, although the indirect associations were lesser in magnitude than the direct association.

**Conclusions:**

This study found a clear association between clinically diagnosed IBS status and interference in work by gastrointestinal symptoms in which sense of coherence might be of importance.

## Introduction

Irritable Bowel Syndrome (IBS) is a chronic, relapsing gastrointestinal disorder characterised by recurrent abdominal pain and disturbed bowel habits [1, 2]. The diagnosis is based on symptoms and exclusion of organic gastrointestinal disease and affects 10–15% of the general population and has a female predominance [3–5]. IBS is subtyped according to the predominant symptoms into constipation (IBS-C), diarrhoea (IBS-D) mixed (IBS-M) and unspecified (IBS-U) [6].

The aetiology of IBS remains unclear but disturbance of function along the brain-gut axis has been proposed [7, 8]. To date, no specific biological abnormality has been identified that could explain the symptoms with specific exceptions such as post-infectious IBS [9]. However, well known theories about the pathophysiology of IBS include gastrointestinal dysmotility, visceral hypersensitivity, low-grade inflammation, increased intestinal mucosal permeability, immunological and genetic factors as well as altered intestinal microbiota [9–13]. IBS is also associated with psychosocial factors such as impaired quality of life, comorbid psychiatric disorders, chronic life stress and impaired coping. The heterogeneity of pathophysiological and psychosocial factors has led to the concept of a bio-psychosocial disease model in IBS [14–20]. IBS affects adults of all ages and especially those of working ages [21], often resulting in gastrointestinal problems that affect daily performance and productivity in working life, and lead to a greater extent of short periods of sick leave and impaired health-related quality of life (HRQL) [16, 22, 23]. Dibonaventura et al. showed that reduced productivity while at work in IBS-C patients was important as a contributor to total reduced work productivity, and that IBS-C patients experienced even more reduction in work productivity than a comparison group without IBS [24]. Faecal incontinence (FI) may also play a role in work impairment a recent study showed more impairment in IBS patients with comorbid FI [25].

Individuals use different coping strategies to manage illness and stress, which is also relevant for those affected by IBS [26, 27]. These coping strategies can have positive effects, but might also have negative effects on their health status. Coping strategies using avoidant behaviour are characterised by a tendency to escape rather than to manage difficulties. This behaviour is related to an increase in self-blame and could lead to poor psychological adjustment i.e. lower coping ability [26]. A recent study using Sense of coherence (SOC) showed inferior coping strategies in IBS patients compared to non-IBS patients [17]. Drossman and co-workers demonstrated in patients with IBS that illness behaviour was the strongest predictor of the severity of functional bowel disorders [27].

To our knowledge, few studies have been conducted that examine the association between SOC and work productivity in IBS patients. We therefore conducted this study with the aim of investigating the role of Sense of coherence in work productivity among patients diagnosed with IBS compared with patients without any present gastrointestinal complaints in a primary care setting. We hypothesised that individuals with IBS might have inferior coping strategies and less confidence in the public health system, which might both directly and indirectly reduce their productivity in working life.

## Material and methods

### Study population and design

The study adopted a case-control design focussing on patients diagnosed with IBS, i.e. IBS cases in a defined region in south-east Sweden (The County Council of Östergötland). Ten Primary Healthcare centres (PHCs), in the three major cities of the region joined the study. These PHCs are responsible for primary care of a population of around 150,000 inhabitants (1/3 of the entire population of the county) [28]. The selected ten PHCs were chosen based on defined criteria to ensure diversity concerning socioeconomic status, age of population and number of immigrants. Subjects within the normal working age (range 18–65 years) with a known IBS diagnosis, diagnosed by a physician and active symptoms during the last two years identified in the patient medical register of the selected PHC were invited to participate in the study. The control group comprised other patients at these healthcare centres with a similar age and sex distribution, who sought care for other but less serious complaints not associated with GI symptoms and with no GI diagnoses found in the patient register for the previous two years. Patients who agreed to participate after an invitation letter completed questionnaires and returned them by mail in pre-paid envelopes. A total of 1135 invitations were mailed out, of which *n* = 754 individuals agreed to participate, yielding an initial response rate of 66%. However, *n* = 188 individuals who agreed to participate did not return a questionnaire, yielding a final response rate of 50%. A total of *n* = 305 IBS patients agreed to participate in this study, and patients without IBS and any present or previous GI complaints comprised *n* = 369 controls.

### Questionnaire

Questionnaires applied in this study include instruments designed to measure confidence in the social security system and in the community and derives from The Swedish Living Conditions Survey of Health and Welfare Survey [29]. Further, questions were asked about whether gastrointestinal problems might affect working life as well as the Sense of coherence questionnaire and questions to define IBS according to the ROME III criteria [30].

### Sense of coherence (SOC)

Sense of coherence is a theoretical construct explaining differences in how people perceive the world around them and thereby how they tend to cope with stressful and strenuous situations. This concept includes three main components: comprehensibility (the ability to understand what happens), manageability (to what extent the person was able to manage the situation) and meaningfulness (the ability to find meaning in the situation) [31]. This concept has been suggested as explaining how individuals cope with stressors in their lives. The higher the score, the more effective their coping strategy and the better their health outcome. The Swedish version of Antonovsky’s 13-item questionnaire (SOC-13) was used in this study [32]. Every item is scored on a Likert scale ranging from 1 to 7 points. Thus, the total score ranges from 13 to 91 points. This 13-item version of SOC has been shown to be reliable, valid and cross-culturally applicable when evaluating how well people can manage stress and still be healthy [33].

Education was divided into three categories: low (primary school), medium (secondary or upper secondary school), or high (college or university). Marital status was dichotomised into the categories: 1) living alone or 2) married or living together. Occupational status was divided into five categories: employed, unemployed, retired, on sick-leave or student. Questions about whether gastrointestinal problems affect daily performance in working life offered four possible responses: yes absolutely, yes partly, no it does not affect my daily work, no I have no gastrointestinal problems. Questions about confidence in the healthcare system offered five possible responses: great confidence, quite a lot of confidence, not very much confidence, no confidence, and have no idea.

### Statistical analyses

**Fig. 1:**
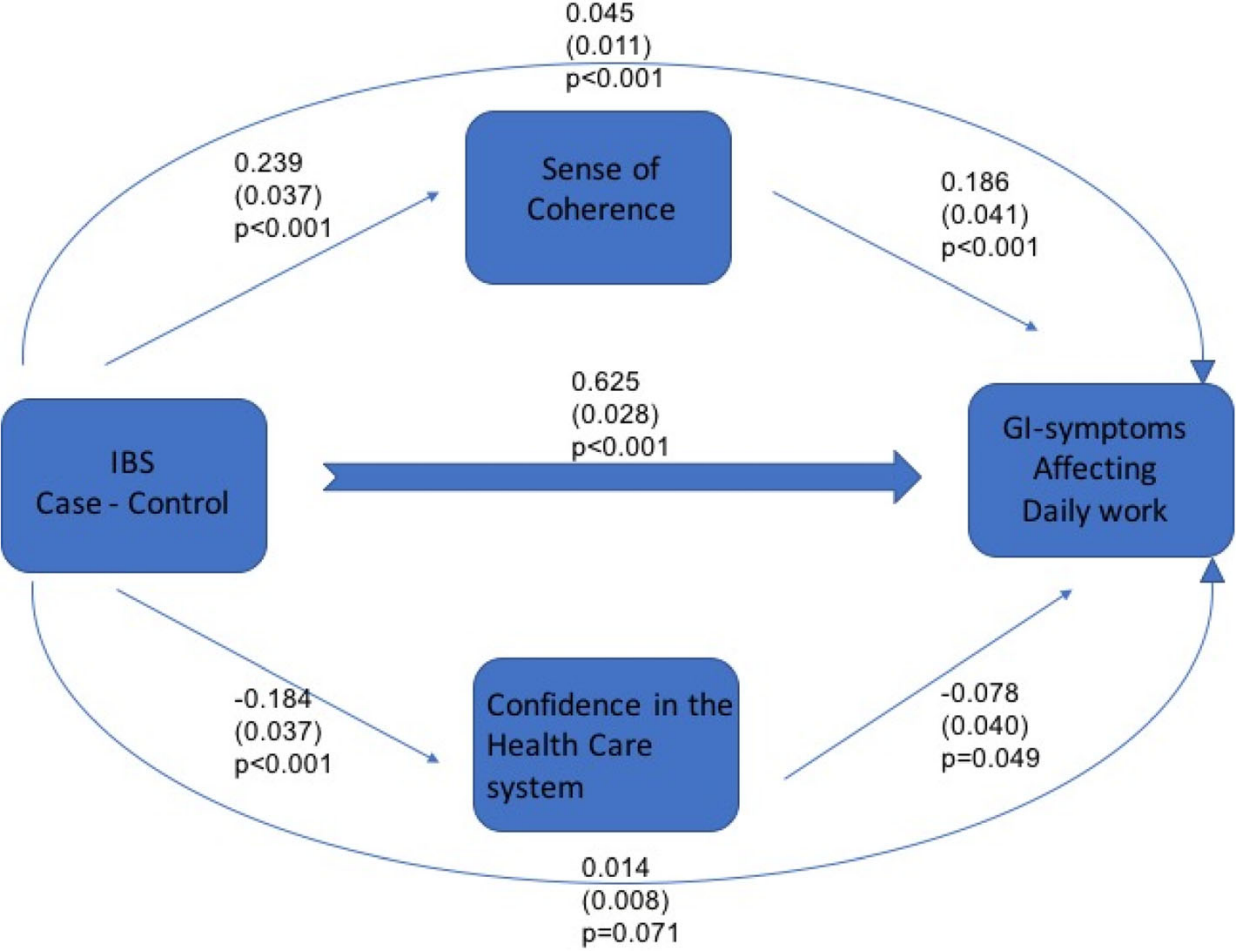
Analytic framework for the study - A model of potential direct and indirect factors affecting daily performance at work

The design of the analysis in this study can be summarised according to the model shown in Fig. 1. To examine whether the association between IBS status and interference with work by gastrointestinal symptoms might operate indirectly via either Sense of coherence or confidence in the healthcare system or not, an a priori path model was fitted, as described in Fig. 1. If the indirect paths in the model account for a substantial fraction of the association, there is a possibility that Sense of coherence or confidence in the healthcare system are important in explaining the association between IBS and interference. Since the model is being estimated from a cross-sectional design it is important to note that causal interpretations cannot be made from it but the findings are an important first step that should be followed up using a longitudinal design. Due to violation of the assumption of multivariate Normality, formal statistical inference (calculation of standard errors and hypothesis testing) has been undertaken using the nonparametric bootstrap. All path coefficients (measures of association) are report in standardised form to facilitate comparability. Model fit is not directly relevant to the research question, so it is not reported. The key metric is the fraction of the total association between IBS and interference with work is indirectly via Sense of coherence and confidence in the health system. Quantitative variables are described using mean and standard deviation (s.d.) while qualitative (categorical) variables are described using counts and percentages. Comparisons between IBS cases and controls were undertaken using Pearson Chi-Square tests for quantitative variables and qualitative variables.

## Results

For the total sample (*N* = 668 IBS cases and controls) there was a predominance of females for both groups and the mean age was significantly higher (*p* <  0.0001) for IBS cases 46.7 (s.d. 13.0) compared to 51.4 years (s.d. 11.8) for controls. Regarding psychosocial environment, the IBS cases significantly (*p* < 0.0001) tended to live alone more, and had a lower education level (*p* = 0.002) than the control group. IBS cases and controls did not differ significantly with respect to sex-ratio, born abroad or occupational status, as shown in Table 1.

**Table 1:** Sociodemographic factors for IBS cases and Controls

	IBS cases (*n* = 299)		Controls (*n* = 369)		*p*-value
Gender	n	%	n	%	0.18
Male	65	22.0	65	18.0	
Female	234	78.3	304	82.4	
Marital status					< 0.0001
Living alone	82	28.0	62	17.0	
Married/cohabitant	215	72.4	306	83.2	
Education					0.002
Low	51	17.1	32	9.0	
Medium	136	46.0	164	45.0	
High	111	37.2	171	47.0	
Occupational status					0.31
Employed	208	70.0	278	75.3	
Student	23	8.0	18	5.0	
Retired	40	14.0	47	13.0	
Long - term sick-leave	13	4.4	9	2.4	
Unemployed	13	4.4	17	5.0	
Born abroad	29	10.0	27	7.3	0.26
Age mean	46,7		51,4		< 0.0001
(s.d.)	13.0		11.8		

Statistically significant differences are reported in Table 2 between IBS cases and controls concerning abdominal pain or discomfort affecting everyday performance at work (*p* < 0.0001) as well as chanced work due to abdominal problems (*p* = 0.007). IBS cases also showed significantly lower (*p* = 0.001) confidence in public health than the control group. Average score on Sense of coherence was also significantly lower (*p* < 0.0001) among IBS cases compared to controls, see Table 2.

**Table 2:** Factors related to IBS and work

	IBS cases (*n* = 299)		Controls (*n* = 369)		*P*-value
Factors:	n	%	n	%	
Abdominal symptoms affect performance at work	167	78.4	51	18.0	< 0.0001
Changed work due to abdominal problem	11	5.2	2	0.9	0.007
Confidence in healthcare					0.001
Yes	213	72.0	307	83.4	
Very little or none	82	28.0	60	16.3	
Do not know	3	1.0	1	0.3	
Sense of coherence mean	61.3		67.0		< 0.0001
(s.d.)	11.6		10.5		

Figure 1 implements an a priori identified model of associations between clinically diagnosed IBS status and interference in work by gastrointestinal symptoms. Of primary interest is the direct versus indirect (via Sense of coherence and confidence in the healthcare system) associations between IBS status and interference. The total indirect association between IBS status and interference (b = 0.059, SE = 0.016) is small relative to the direct association (b = 0.625, SE = 0.028), but statistically significant. The indirect association via sense of coherence (*p* = 0.045) is substantially larger and more clearly statistically significant than via confidence in the healthcare system (*p* = 0.014), as shown in Fig. 1. The analytic model shown in Fig. 1 turns out that the correlation between SOC total score and the healthcare seeking is modest and negative (r = − 0.2). (This is not shown in the figure).

## Discussion

The negative effects of IBS on everyday working activity have been discussed earlier in the literature [16, 22, 24]. Nevertheless, few studies have focussed on potential direct or indirect factors associated with impaired work productivity among IBS patients. The results of this study add to our understanding of the psychosocial complexity of IBS. The theoretical model that underpins our hypotheses is primarily the biopsychosocial model of George Engel [34], applied to IBS specifically by Doug Drossman [20, 35, 36]. This model allows the social, psychological and behavioural dimensions of illness. Using the concept of the biopsychosocial model might be a good way to explain the interaction between psychological and physiological factors which is often expressed in IBS patients. Our main result in the present study is an association between clinically diagnosed IBS status and interference in work by gastrointestinal symptoms. Of primary interest are the direct versus indirect associations via sense of coherence and confidence in the healthcare system between IBS status and interference in everyday working impairment.

Our primary finding is that both Sense of coherence and confidence in the healthcare system are involved in the association between IBS status and GI symptoms affecting an individual at work. Of further interest is that sense of coherence, which we interpret as a coping strategy, appears to be more important in the association than confidence in the healthcare system. More maladaptive coping strategies have also been shown to be an important factor in previous studies. Our work now suggests it may be part of the mechanism through which IBS has an impact in important aspects of people’s lives. Further, improving the coping strategies of IBS patients may therefore improve the quality of life of IBS individuals and help to increase economic productivity.

Our previous research also showed that gastrointestinal symptoms affect daily performance in working life in IBS patients and especially female IBS patients reported more frequent short and long-term sick leave due to GI problems than their controls [16] but we were unable to indicate why, until now. Other studies have also reported that IBS patients miss approximately one or two working days per month due to their disease [22, 37]. There may be many explanations for this observed association, including that IBS symptoms may reduce the self-estimated fitness to work through different reasons, including embarrassment when using public toilets, intense abdominal pain, urgency to reach a toilet, fear of faecal soiling [37], faecal incontinence [25] or fatigue [38, 39]. On the other hand, few IBS patients in the present study reported that they had changed their employment due to gastrointestinal problems. Nevertheless, many IBS patients develop personal coping strategies to manage this kind of problem [26, 27]. Both short- and long-term sick leave might be one way to cope with the illness. Our previous study pointed out that long-term sick leave is seen more frequently among IBS patients compared to non-IBS individuals [16]. Our new data suggests that greater use of sick leave may be a reflection of maladaptive coping strategies by IBS patients and this finding is in concordance with consensus from other publications in this field [40–42].

Findings in the present study seem somewhat contradictory, because IBS patients reported lower confidence in the public health system than their controls did, but apparently, they utilise healthcare. Of course, the reported low confidence may be due to extensive use of the healthcare system without cure, which has led to low confidence. Our data are also in concordance with other studies showing that patients with IBS have an increased use of healthcare resources, visit the doctor more frequently, use more diagnostics tests and consume a larger amount of medications than those without IBS [23]. If an individual does not feel the confidence of the community or its representatives, it is unlikely that the individual will seek help when it is needed, potentially leading to more serious health consequences. Unfortunately, we have no data to suggest why IBS healthcare seekers have lower confidence in the public healthcare system than non-IBS healthcare seekers. Another explanation could be that IBS patients simply have more negative experiences with their contact with these institutions. Maybe it reflects inferior coping ability or, as reported in other studies, that IBS patients tend to have more negative early life events such as physical, sexual an emotional abuse [43, 44]. These factors could contribute to less confidence in the community in general, and its support systems in particular.

Our study had both strengths and limitations. One strength is that we used established and validated questionnaires. A possible limitation of using IBS diagnoses made in a PHC (as we have done), is the dependence on the general practitioners’ ability to make the correct diagnosis. On the other hand, it could also be a strength, because most IBS patients are diagnosed in primary care. Other strengths are that our selection of a control group, which in this case consists of patients without GI problems, enables us to find associations to IBS itself. Another possible limitation could be the use of self-reported data from questionnaires. A well-known phenomenon to take into consideration when using self-reported data is recall bias, but in general, self-reports are quite reliable and well established [45]. However, what was beyond the scope of the study design was to analyse direct and indirect cost for employers and employees.

## Conclusion

An a priori model shows association between clinically diagnosed IBS status and interference in work by gastrointestinal symptoms. Associations between IBS status and interference were also seen, both direct and indirect via Sense of coherence and confidence in the healthcare system, with sense of coherence appearing to be particularly important. Finally, from IBS patients’ and employers’ point of view, efforts to treat IBS patients in primary healthcare with cognitive behaviour therapy, by educating patients about IBS, or by learning to cope with the disease might be beneficial for both groups and might also increase the HRQL among this patient group.

## Data Availability

The datasets generated and/or analysed during the current study are not publicly available, but are available from the corresponding author on reasonable request.

## Abbreviations

FI: Fecal incontinence
GI: Gastrointestinal
HRQL: Health related quality of life
IBS: Irritable bowel syndrome
IBS-C: Irritable bowel syndrome with constipation
IBS-D: Irritable bowel syndrome with diarrhoea
IBS-M: Irritable bowel syndrome mixed
IBS-U: Irritable bowel syndrome unspecified
PHC: Primary Healthcare Centre
SE: Standard error
SOC: Sense of coherence
